# Reduced intestinal‐to‐diffuse conversion and immunosuppressive responses underlie superiority of neoadjuvant immunochemotherapy in gastric adenocarcinoma

**DOI:** 10.1002/mco2.762

**Published:** 2024-10-28

**Authors:** Lei Wang, Linghong Wan, Xu Chen, Peng Gao, Yongying Hou, Linyu Wu, Wenkang Liu, Shuoran Tian, Mengyi Han, Shiyin Peng, Yuting Tan, Yuwei Pan, Yuanfeng Ren, Jinyang Li, Haihui Wen, Qin Liu, Mengsi Zhang, Tao Wang, Zhong‐Yi Qin, Junyu Xiang, Dongfeng Chen, Xianfeng Li, Shu‐Nan Wang, Chuan Chen, Mengxia Li, Fan Li, Zhenning Wang, Bin Wang

**Affiliations:** ^1^ Department of Gastroenterology & Chongqing Key Laboratory of Digestive Malignancies Daping Hospital Army Medical University (Third Military Medical University) Yuzhong District Chongqing China; ^2^ The 904th Hospital of the Joint Logistics Support Force of the Chinese People's Liberation Army Changzhou China; ^3^ School of Medicine Chongqing University Chongqing China; ^4^ Department of Surgical Oncology and General Surgery The First Hospital of China Medical University, Key Laboratory of Precision Diagnosis and Treatment of Gastrointestinal Tumors, Ministry of Education, China Medical University Heping District Shenyang China; ^5^ Department of Pathology Daping Hospital Army Medical University Chongqing China; ^6^ Department of Medicine Ocean University of China Qingdao China; ^7^ Institute of Pathology and Southwest Cancer Center Key Laboratory of Tumor Immunopathology of Ministry of Education of China Southwest Hospital Army Medical University (Third Military Medical University) Chongqing China; ^8^ Department of Radiology Daping Hospital Army Medical University Chongqing China; ^9^ Cancer Center of Daping Hospital Army Medical University Chongqing China; ^10^ Gastric and Colorectal Surgery Division Department of General Surgery Daping Hospital Army Medical University Chongqing China; ^11^ Jinfeng Laboratory Chongqing China

**Keywords:** gastric adenocarcinoma, immunochemotherapy, Lauren's classification, neoadjuvant therapy, tumor immune microenvironment

## Abstract

Neoadjuvant immunochemotherapy (NAIC) achieves superior clinical benefits over neoadjuvant chemotherapy (NAC) in multiple types of human cancers, including gastric adenocarcinoma (GAC). However, it is poorly understood how the malignant epithelial cells and tumor immune microenvironment (TIME) might respond distinctly to NAIC and NAC that underlies therapeutic efficacy. Here treatment‐naive and paired tumor tissues from multiple centers were subjected to pathological, immunological, and transcriptomic analysis. NAIC demonstrated significantly increased rate of pathological complete response compared to NAC (pCR: 25% vs. 4%, *p *< 0.05). Interestingly, pretreatment intestinal subtype of Lauren's classification was predictive of pathologic regression following NAIC, but not NAC. A substantial portion of cancers underwent intestinal‐to‐diffuse transition, which occurred less following NAIC and correlated with treatment failure. Moreover, NAIC prevented reprogramming to an immunosuppressive TIME with less active fibroblasts and exhausted CD8^+^ T cells, and increased numbers of mature tertiary lymphoid structures. Mechanistically, activation of the tumor necrosis factor alpha (TNFα)/nuclear factor‐kappa B (NF‐κB) signaling pathway was associated with response to NAIC. Together, NAIC is superior to NAC for locally advanced GAC, likely due to reduced intestinal‐to‐diffuse conversion and reprogramming to an immuno‐active TIME. Modulation of the histological conversion and immunosuppressive TIME could be translatable approaches to improve neoadjuvant therapeutic efficacy.

## Graphical abstract

The intestinal subtype of Lauren's classification can improve the efficacy of NAIC by preventing reprogramming to immunosuppressive cells, reducing activated fibroblasts and exhausted CD8+ T cells, inducing the formation and maturation of T‐cell‐rich inflamed areas, and activating the TNFα signaling pathway through NF‐κB.

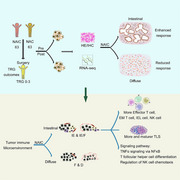

## 1 INTRODUCTION

Gastric adenocarcinoma (GAC) is one of the most prevalent cancers around the world and a leading cause of cancer‐associated death, especially in east Asian countries.^1^, ^2^ A significant proportion of GAC patients receive diagnoses at advanced stages, resulting in an overall survival rate of less than 50% within 5 years.^3^ For these patients, neoadjuvant chemotherapy (NAC) significantly improves their clinical benefits. The MAGIC study was the first to show that NAC is superior to surgery alone in pathological regression and overall survival, establishing the foundation of NAC for GAC,^4^ which was subsequently confirmed by the FLOT4‐AIO and RESOLVE studies.^5^, ^6^ Therefore, NAC plus D2 gastrectomy was recommended as standard treatment modality for locally advanced GAC by multiple guidelines.^7^, ^8^ However, most patients who received NAC did not survive past 5 years, indicating an urgent need to improve neoadjuvant therapies.

Neoadjuvant immunochemotherapy (NAIC) with immune checkpoint inhibitors (ICIs) plus chemotherapy may be a promising pre‐operative approach. For those patients with unresectable/metastatic GAC, combinatory application of chemotherapy and ICIs significantly improved clinical outcomes in multiple phase III clinical trials including CheckMate‐649^9^ and KEYNOTE‐811.^10^, ^11^, ^12^, ^13^ Moreover, in a phase II PANDA trial, researchers evaluated the efficacy of PD‐L1 inhibitor atezolizumab plus chemotherapy in neoadjuvant therapy for local advanced gastric or gastroesophageal junction (GEJ) adenocarcinoma. Out of the 20 included patients, 14 (70%) achieved major pathological regression (MPR) following treatment, with nine (45%) achieving pathological complete regression (pCR).^14^ Indeed, the favorable antitumor effects of NAIC in locally advanced GAC is being reported in several prospective phase II clinical trials,^15^, ^16^, ^17^ with the pCR rates ranging from 19.4% to 33.3%. These preliminary studies suggested that NAIC may provide improved benefits for locally advanced GAC, while further clinical evidence to support these notion is needed and warrants a direct comparison of the therapeutic efficacies of NAIC and NAC.

Dynamic remodeling of both cancer cells and the tumor immune microenvironment (TIME) significantly influences responses to chemotherapy in patients with GAC.^18^ This phenomenon remains consistent in the context of NAIC, as evidenced by the reprogramming of tumor subclones, remodeling of the immune microenvironment, and alterations in the T‐cell receptor (TCR) repertoires, irrespective of the pathological response.^19^ A recent study employed unsupervised clustering of RNA‐seq data to classify the TIME into four subtypes based on the composition of immune cells and stromal cells.^20^ These subtypes have been shown to predict the efficacy of ICIs in a variety of cancers, including gastric, bladder, and melanoma. Among these subtypes, the “immune‐enriched, non‐fibrotic” subtype showed the highest response rate, while the “fibrotic” subtype exhibited the lowest response rate across different types of cancers. Tumor‐infiltrating immune cells and intratumor microbiome have a significant impact on tumor progression and responses to anticancer therapies, thereby supporting this observation.^21^, ^22^, ^23^ However, it remains unknown how NAIC and NAC may differentially impact the malignant epithelial cells and TIME in GAC. The immunophenotype suitable for immunotherapy, particularly NAIC, remains unclear. A comprehensive analysis of the epithelial and TIME alterations between the two neoadjuvant regimens is warranted to provide insights into how they might elicit therapeutic responses in humans.

Here, analysis of multi‐cohort clinical data revealed that NAIC induced significantly higher rate of pCR compared to NAC in patients with locally advanced GAC. Through histological, immunological, and transcriptomic analysis of the pre‐ and post‐treatment samples, we identified dynamic alteration of histological features and TIME in response to neoadjuvant therapies. Notably, pretreatment intestinal phenotype was associated with pathologic regression in patients receiving NAIC, where intestinal‐to‐diffuse transition was less frequently observed in NAIC compared to NAC. Moreover, NAIC inhibited reprogramming of the TIME to be immunosuppressive, thus leading to increased neoadjuvant therapeutic efficacy.

## 2 RESULTS

### 2.1 Patients and preoperative treatment

**FIGURE 1:**
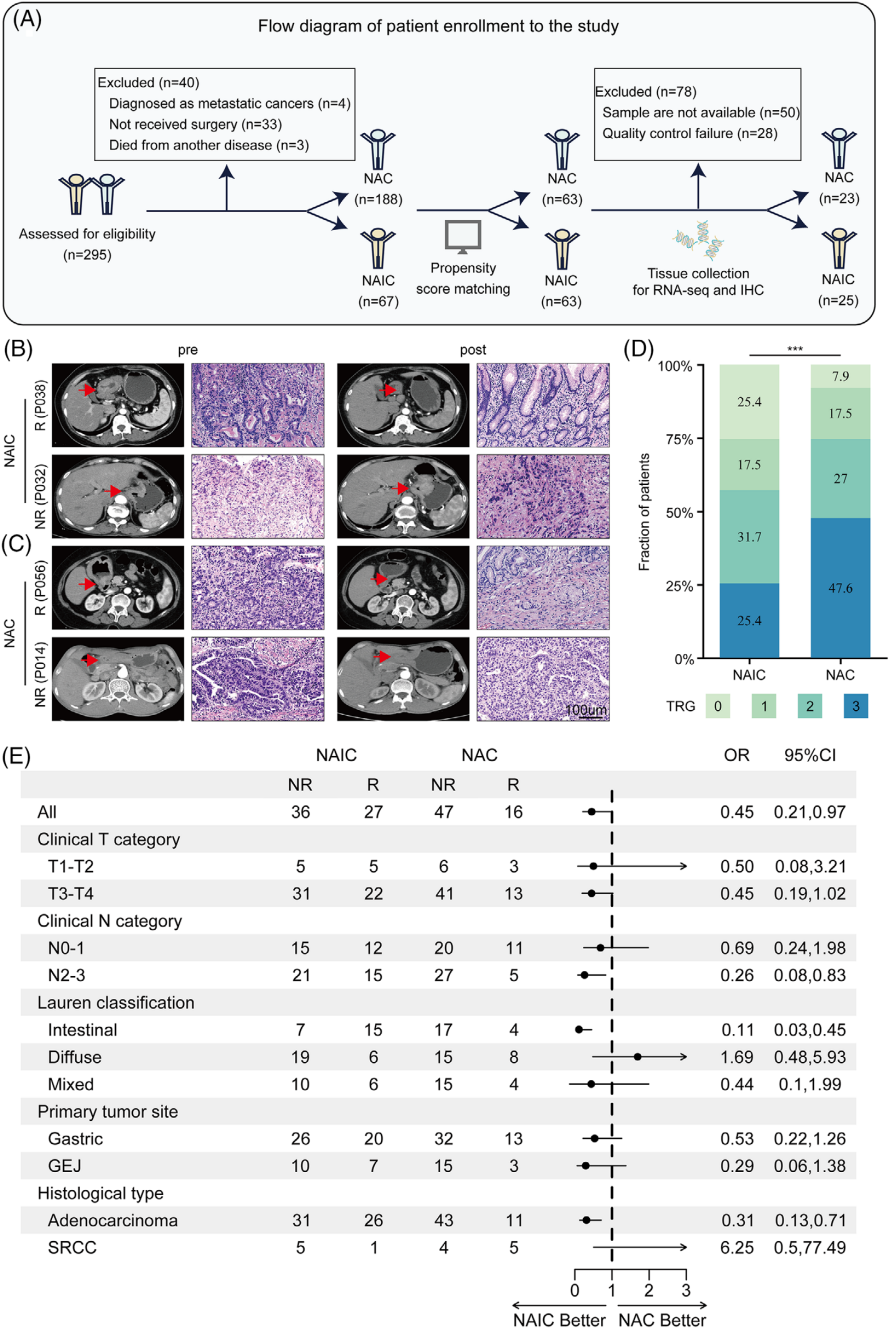
Clinical efficacy and treatment effect on tumor regression grade by subgroups in neoadjuvant immunochemotherapy and chemotherapy in gastric adenocarcinoma. (A) Flow diagram of study design. (B) Representative radiological and pathological in responders (top, P038) and non‐responders (bottom, P032) in the neoadjuvant immunochemotherapy group. Red arrow: the location of tumor lesions. (C) Representative radiological and pathological in responders (top, P056) and non‐responders (bottom, P014) in the neoadjuvant chemotherapy group. Red arrow: the location of tumor lesions. (D) Comparison of tumor regression grade between neoadjuvant immunochemotherapy (NAIC) and neoadjuvant chemotherapy (NAC). (E) Treatment effect on tumor regression grade by subgroup. 95% CI, 95% confidence internal; ECOG, Eastern Cooperative Oncology Group; GEJ, gastroesophageal junction; IHC, immunohistochemistry; NGS, next‐generation sequencing; OR, odds ratio; SRCC, signet‐ring cell carcinoma; TRG, tumor regression grade.

As shown in Figure 1A, after performing propensity score matching (PSM), a total of 126 patients were enrolled, including 63 patients who received NAIC and 63 patients who received NAC. There were no significant differences in baseline clinical characteristics between the two groups (Table S1). Among the NAIC cohort, the median age of patients was 62 years (range: 54−68), and 41 (65%) patients were male. In terms of Lauren classification, 22 (35%) had intestinal type, 25 (40%) had diffuse type, and 16 (25%) had mixed type. The median age of the NAC group was 61 years (range: 56−67), and 43 (68%) patients were male. Among the patients, 21 (33%) had intestinal type, 23 (37%) had diffuse type, and 19 (30%) had mixed type.

### 2.2 Neoadjuvant immunochemotherapy achieves a higher pCR rate and comparable side‐effects compared to chemotherapy

All patients underwent D2 gastrectomy after the completion of neoadjuvant therapy. Among the NAIC group, 32 (50%) patients underwent distal gastrectomy, five (8%) patients underwent proximal gastrectomy, and 26 (42%) patients received total gastrectomy. Sixty‐three (100%) patients achieved R0 resection in the NAIC group and 62 (98%) patients achieved R0 resection in the NAC group. As for the pathological evaluation of primary tumor, 17 (27%) patients achieved ypT0 in the NAIC group, while five (8%) patients in the NAC group. With regard to Lauren's classification after neoadjuvant therapy, 10 (16%) patients had intestinal histological type, 21 (34%) patients had diffuse histological type, and 16 (25%) patients had the mixed type in the NAIC group, while in the NAC group, nine (14%) patients had intestinal histological type, 35 (56%) patients had diffuse histological type, and 14 (22%) patients had the mixed type. The representative computed tomography images and hematoxylin and eosin (H&E) staining micrographs of pathologic response (responder or non‐responder) in patients before and after NAIC or chemotherapy are shown in Figure 1B,C.

In the NAIC group, a higher proportion of pCR (TRG 0) was observed (16 patients, 25%), while only five patients (8%) achieved pCR in the NAC group (Figure 1D). Similar results were observed for MPR (TRG 0/1), with 27 patients (43%) achieving MPR in the NAIC group and 16 patients (25%) in the NAC group (Figure 1C). Overall, the NAIC group showed a significantly better pathological response compared to the NAC group (details provided in Table S2).

In exploratory subgroup analyses of tumor regression grade (TRG), compared with the NAC group, all the parameters favored the NAIC group, except for the diffuse type of Lauren's classification and the histologic type of signet ring cell carcinoma (Figure 1E). In the intestinal subgroup, 15 (68.2%) patients in the NAIC achieved a response compared to four (19%) in the NAC, with an odds ratio (OR) for TRG at 0.11 (95% confidence interval [CI] 0.03‒0.45). Subgroup analyses by clinical N category suggested a TRG benefit for NAIC compared with NAC in the N2‐3 subgroup, with an OR for TRG at 0.26 (95% CI 0.08‒0.83). Additionally, in the adenocarcinoma subgroup, NAIC had significantly higher response rates than NAC (45.6% vs. 28.9%; OR 0.31; 95% CI 0.13‒0.71) (Figure 1E).

The incidence of any grade TRAEs in NAIC group and NAC group was comparable (95.2% and 96.8%). The incidence of grade 3 or greater TRAEs of NAIC group and NAC group were 27.0% and 30.2%, respectively. The most common TRAEs were neutropenia (47.6% in NAIC group vs. 46.0% in NAC group), weight lost (47.6% vs. 49.2%), nausea (42.9% vs. 47.6%), leukopenia (33.3% vs. 34.9%), thrombocytopenia (23.8% vs. 22.2%), anemia (19.0% vs. 20.6%), peripheral neuropathy (12.7% vs. 12.7%), and vomiting (11.1% vs. 6.3%). Most of the TRAEs were grade 1 or 2. The any‐grade immune‐related TRAEs observed in our study were hypothyroidism (3.2%), hypopituitarism (3.2%), hyperthyroidism (1.6%), myocarditis (1.6%), and rash (1.6%). Table S3 demonstrates the details of TRAEs. Overall, NAIC did not increase the incidence of TRAEs compared with chemotherapy alone, and no new toxic adverse events were observed in our study. The TRAEs of NAIC group were well tolerated.

### 2.3 Pretreatment intestinal subtype in histology is predictive of improved pathologic response to neoadjuvant immunochemotherapy which induces less intestinal‐to‐diffuse transition

**FIGURE 2:**
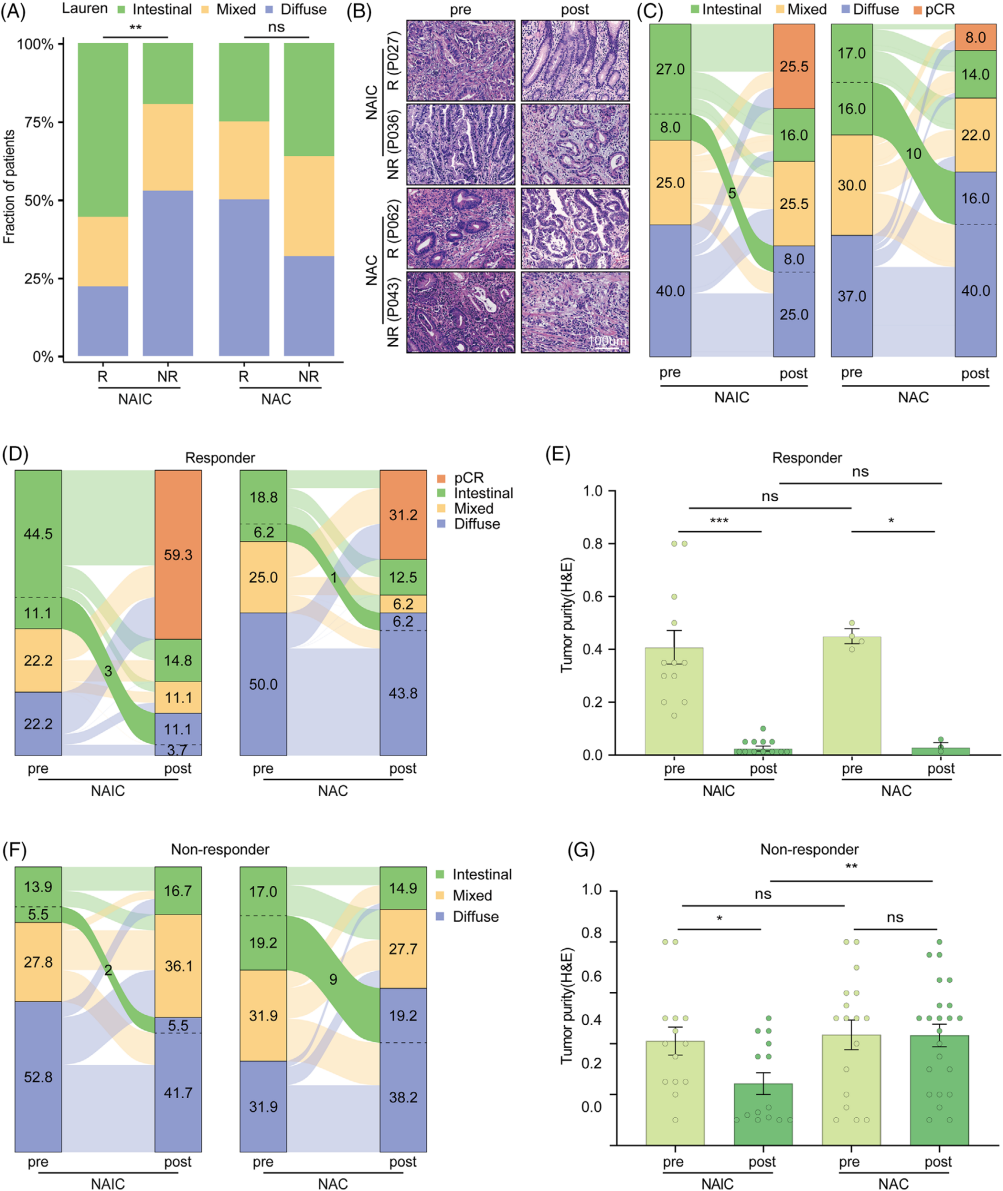
Dynamic alterations of Lauren's classification following neoadjuvant therapies and their correlation to treatment efficacy. (A) Comparison of Lauren's classification between responder and non‐responder groups of different neoadjuvant treatment regimens. (B) Representative hematoxylin and eosin (H&E) staining sections of tumor specimens at pre‐treatment (left) and post‐treatment (right) in neoadjuvant immunochemotherapy (NAIC; top, responder: P027 and non‐responder: P036) and neoadjuvant chemotherapy (NAC; bottom, responder: P062 and non‐responder: P043). (C) Sankey diagram showing the changes of Lauren's classification at pre‐ and post‐treatment in different neoadjuvant treatment regimens: immunochemotherapy (left) and chemotherapy (right). The number of samples underwent intestinal‐to‐diffuse conversion is indicated on the green line. (D) Changes in Lauren's classification pre‐ to post‐treatment in different neoadjuvant treatment regimens: immunochemotherapy (left) and chemotherapy (right) in the responder group. The number of samples underwent intestinal‐to‐diffuse conversion is indicated on the green line. (E) Box plots showing percentages of tumor purity as determine by pathologist on H&E staining in the responder group. (F) Changes in Lauren's classification pre‐ to post‐treatment in different neoadjuvant treatment regimens: immunochemotherapy (left) and chemotherapy (right) in the non‐responder group. The number of samples underwent intestinal‐to‐diffuse conversion is indicated on the green line. (G) Box plots showing percentages of tumor purity as determine by pathologist on H&E staining in the non‐responder group. DGC, diffuse gastric cancer; IGC, intestinal gastric cancer; NR, non‐responder; pCR, pathological complete response; R, responder.

Subgroup analysis of the Lauren's classification revealed that the intestinal‐type gastric cancer achieved better pathological regression following NAIC but not NAC, while the diffuse subtype is more resistant to NAIC (Figure 1E). The quantitative data and representative H&E staining images of Lauren's classifications in patients following NAIC or NAC are shown in Figure 2A,B. However, there was no obvious correlation between the clinical efficacy of Lauren's classification and NAC. It appears that pretreatment intestinal type of Lauren's classification of GAC could be a potential biomarker specifically for NAIC but not NAC.

Moreover, analysis of the pathological status of paired tissue samples before and after neoadjuvant therapies revealed that a substantial portion of cancers underwent intestinal‐to‐diffuse transition following treatment (Figure 2C). The intestinal‐to‐diffuse conversion was less likely observed in the NAIC group, as compared to the NAC group (NAIC vs. NAC, 8.0% vs. 16.0%; Figure 2C). As a result, the proportion of diffuse subtype histology increased significantly after NAC (pre vs. post, 37.0% vs. 56.0%), while the diffuse subtype cancers decreased slightly following NAIC (pre vs. post, 40.0% vs. 33.0%).

We assessed whether the histological transition was associated with the clinical efficacy of neoadjuvant treatment. In the responder group which achieved higher pathological remission, only a few cases displayed features of histological changes (Figure 2D). There were no significant differences among the two groups, thus leading to comparable proportion of diffuse subtype with similar decreases in the tumor purity (Figure 2D,E). However, in the non‐responders, more intestinal‐to‐diffuse conversion was identified in the NAC group, which resulted in significantly increased proportion of diffuse subtype after NAC but not NAIC (NAIC vs. NAC, 5.5% vs. 19.2%; Figure 2F,G). Thus, NAIC induces less intestinal‐to‐diffuse conversion compared with NAC, which might be an underlying reason why it achieves higher therapeutic efficacy.

### 2.4 Neoadjuvant immunochemotherapy induces more immune‐enriched tumor microenvironment as compared to chemotherapy alone

**FIGURE 3:**
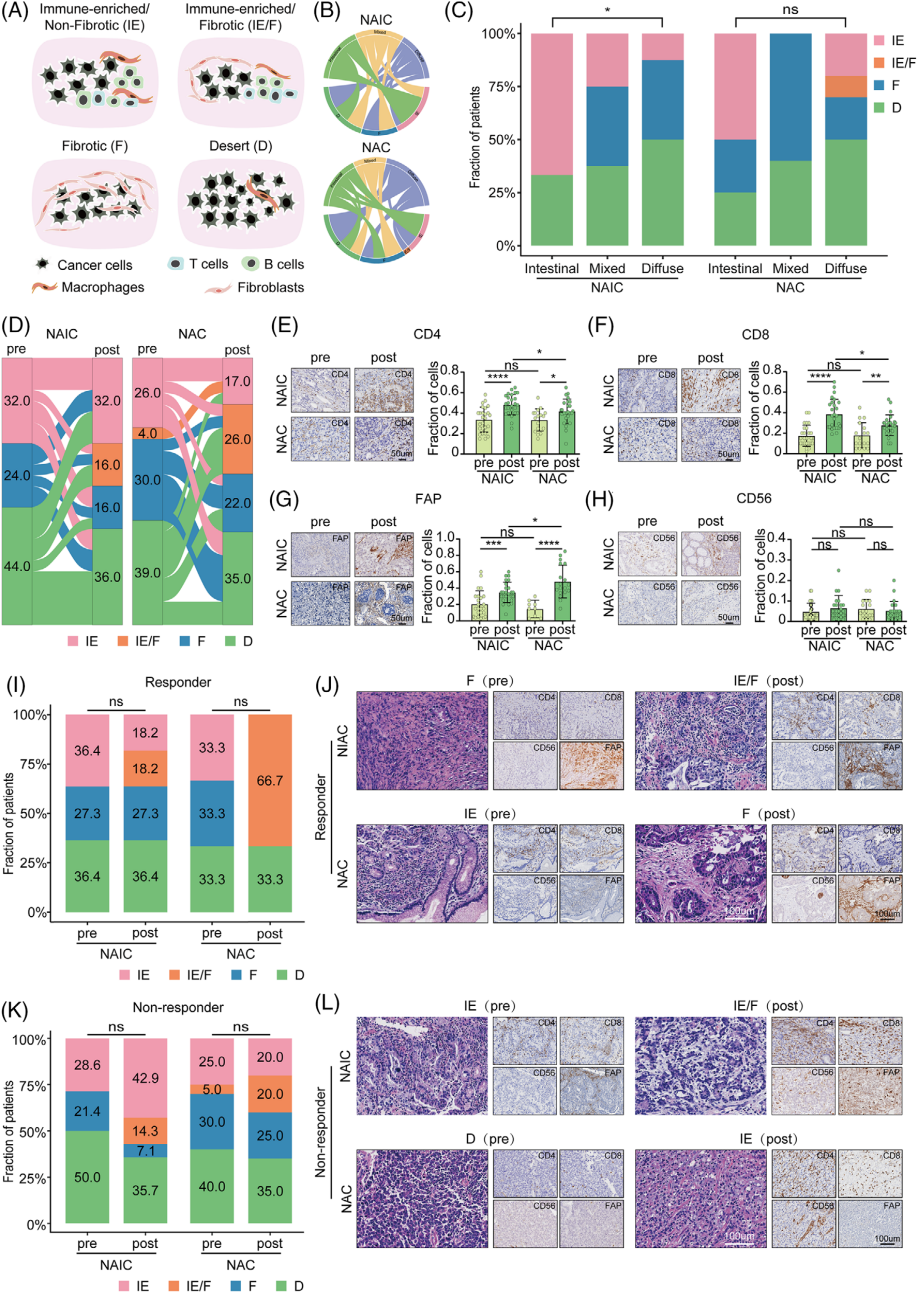
Reprogramming of tumor immune microenvironment (TIME) subtypes and its relation to Lauren's classifications in different neoadjuvant treatment regimens. (A) Putative model of TIME subtype. (B) Circos plot showing the relationship between Lauren's classification and TIME subtypes in neoadjuvant immunochemotherapy (top) and chemotherapy (bottom). (C) Comparison of TIME subtypes among different Lauren's classifications in different neoadjuvant treatment regimens. (D) Sankey plot showing TIME subtypes conversion at pre‐ and post‐treatment in different neoadjuvant treatment regimens: neoadjuvant immunochemotherapy (NAIC; left) and neoadjuvant chemotherapy (NAC; right). (E‒H) Representative images of CD4 (E), CD8 (F), FAP (G), and CD56 (H) staining of tumor specimens pre‐ and post‐treatment with different neoadjuvant treatment regimens (left). Quantification of CD4^+^ T cells, CD8^+^ T cells, activated fibroblast cells, and natural killer (NK) cells by singlet immunohistochemistry and association with pre‐ and post‐treatment with different neoadjuvant regimens (right). (I) Changes in TIME subtypes before and after treatment in different neoadjuvant treatment regimens: NAIC and NAC in the responder group. (J) Representative image of hematoxylin and eosin (H&E) staining of TIME subtypes in the responder group to different neoadjuvant treatment regimens: NAIC (top) and NAC (bottom). Additional staining on right showing associated singlet immunostaining of CD4, CD8, CD56, and FAP. (K) Changes in TIME subtypes before and after treatment in different neoadjuvant treatment regimens: NAIC and NAC in the non‐responder group. (L) Representative image of H&E staining of TIME subtypes in the non‐responder group to different neoadjuvant treatment regimens: NAIC (top) and NAC (bottom). Additional staining on right showing associated singlet immunostaining of CD4, CD8, CD56, and FAP.

TIME can be classified into four subtypes: immune‐enriched/non‐fibrotic (IE), immune‐enriched/fibrotic (IE/F), fibrotic (F), and desert (D), which are predictive of response to tumor immunotherapy (Figure 3A).^20^ We thus defined TIME subtypes of GAC using a K‐nearest neighbor (KNN) model trained by the TCGA‐STAD datasets. Interestingly, the holistic and integrated view identified a tight correlation between Lauren's classification and TIME subtypes at baseline, as visualized by a circos plot (Figure 3B). The plot showed that there were differences in the TIME induced by the Lauren's classification of patients with gastric cancer. The TIME subtypes of intestinal‐type gastric cancer was more likely to be IE subtype, while the TIME of the diffuse gastric cancer was inclined to be F or D subtypes (Figure 3C).

To explore whether the dynamic conversion of TIME subtypes differed between the two neoadjuvant regimens, we employed transcriptomics data to identify TIME subtypes. The results of the study show that NAIC is more likely to induce an IE subtype compared to NAC (32% vs. 17%; Figure 3D). On the contrary, the TIME of patients with locally advanced GAC was more likely to induce IE/F subtype and F subtype after NAC (IE/F: 16% vs. 26%, F: 16% vs. 22%; Figure 3D). Immunohistochemistry (IHC) analysis revealed that two neoadjuvant treatments increased the proportion of CD4^+^ T cells, CD8^+^ T cells, and fibroblasts in the TIME, while the proportion of natural killer (NK) cells did not change significantly before and after neoadjuvant therapy. However, the proportions of CD4^+^ T cells and CD8^+^ T cells after NAIC were significantly higher than that of chemotherapy alone, and the proportion of fibroblasts was significantly lower than that of chemotherapy alone (Figure 3E‒H). These results suggest that NAIC induce more infiltration of immune cells and prevent stromal cell production compared to chemotherapy alone.

Next, we explored the correlation between the TIME and the efficacy of two neoadjuvant therapies. As shown in Figure 3I,J, among patients who received NAIC, the proportion of IE subtype in responder group was higher than that in non‐responder group (36.4% vs. 28.6%). We also obtained similar results in the NAC group (33.3% vs. 25.0%). Meanwhile, the proportion of IE subtypes was higher in patients who underwent NAIC than in those who received chemotherapy alone, whether or not they responded to neoadjuvant therapy. To investigate whether transcriptomics‐based TIME subtypes can be confirmed histologically by assessing the spatial organization of tumor cells, lymphocytes, and stromal cells, we first performed H&E and IHC staining of CD4, CD8, CD56, and FAP on GAC tissues, which labeled CD4^+^ T cells, CD8^+^ T cells, NK cells, and fibroblasts, respectively. It was observed that IHC staining of these specimens are associated with the transcriptomics‐based prediction of TIME subtypes (Figure 3K,L). Overall, TIME subtypes and their dynamic transitions correlate with the efficacy of NAIC. Together, through multi‐omics analysis including transcriptomics and IHC, we demonstrated that NAIC is superior to NAC for locally advanced GAC, likely due to the fact that NAIC induces more immune‐enriched TIME as compared to NAC alone.

### 2.5 Neoadjuvant immunochemotherapy prevents generation of immunosuppressive cells by chemotherapy

To better characterize the dynamic remodeling of TIME by neoadjuvant therapies, a scRNA‐seq dataset was utilized as a reference for deconvoluting bulk RNA‐seq data of pre‐ and post‐treatment samples. A total of 47,511 eligible single cells were categorized into seven different cell types, including immune cells, endothelial cells, and stromal cells. A total of 39 different cell subtypes were identified by further subclustering analysis.

**FIGURE 4:**
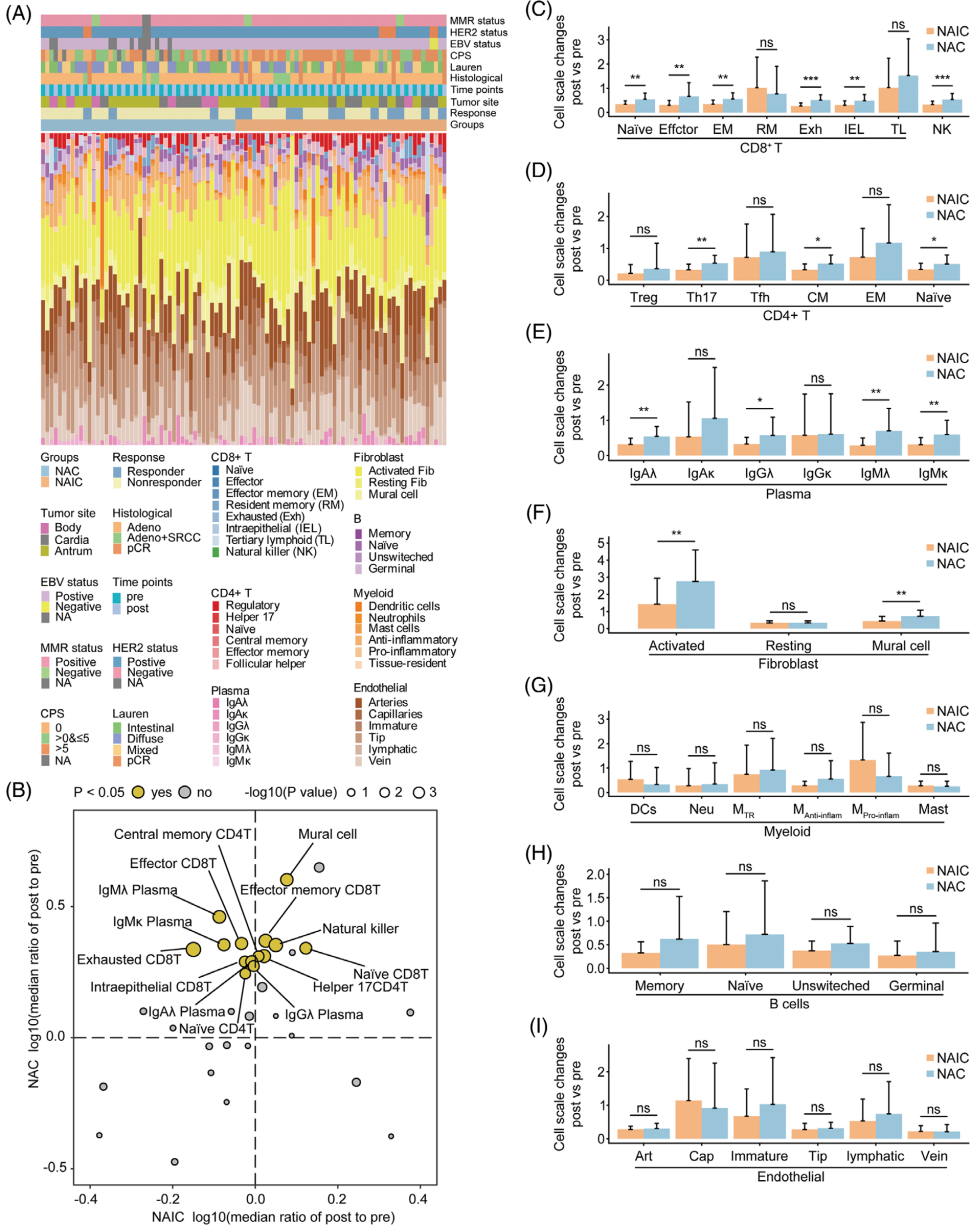
Dynamic changes of immune cell subsets upon neoadjuvant immunochemotherapy and chemotherapy are related to therapeutic responses. (A) Bar charts depicting proportions of seven types include 39 immune cell subtypes estimated by the BayesPrism method based on paired pre‐ and post‐treatment RNA‐sequencing data for each patient. (B) Differential changes in log10(fold change) of immune cell in the deconvolution analysis after two neoadjuvant treatments. Two‐sided Wilcoxon test was conducted to compare the median log10(fold change) of cell populations after neoadjuvant immunochemotherapy (NAIC) and neoadjuvant chemotherapy (NAC) with those before treatment. Yellow dots indicate cell populations in which the change in cell proportion was significantly different (*p* < 0.05) between the two treatment groups. (C‒I) Changes (post‐treatment proportions/pre‐treatment proportions) in different cell subtypes of CD8^+^ T cells (C), CD4^+^ T cells (D), plasma (E), fibroblast (F), myeloid (G), B cells (H), and endothelial (I) under different neoadjuvant treatment regimens.

Next, we examined the abundance and composition of immune, endothelial, and stromal cells in the TIME during neoadjuvant therapy by a BayesPrism deconvolution method (Figure 4A).^24^ Interestingly, a comparison of the changes in cell ratios before and after treatment with the two neoadjuvant regimens revealed that both treatments resulted in increased infiltration of immune cells. However, changes in cell abundance were dramatically different after NAIC compared with NAC alone. We observed significant increases in naive CD8^+^ T cells, effector memory CD8^+^ T cells, NK cells, and Helper17 CD4^+^ T cells, among others, after both neoadjuvant therapies, whereas cell subpopulations such as effector CD8^+^ T cells, exhausted CD8^+^ T cells, naive CD4^+^ T cells, central memory CD4^+^ T cells, IgAλ, IgGλ, IgMλ, and IgMκ were significantly increased only after NAC, but not after NAIC (Figure 4B‒E). Interestingly, although more infiltration of immune cells occurred after NAC, there was an equal increase in infiltration of activated fibroblasts and mural cells (Figure 4F), which is consistent with a dynamic shift in TIME subtypes (Figure 3B). No significant differences were observed between the two neoadjuvant treatments in cell subpopulations of cell types such as myeloid cells, B cells, or endothelial cells (Figure 4G‒I). These data indicate that the superiority of NAIC over NAC alone may be attributed to the fact that NAIC only significantly increased the abundance and content of immune cells in the TIME, while chemotherapy alone increased the proportion of both immune cells and stromal cells.

**FIGURE 5:**
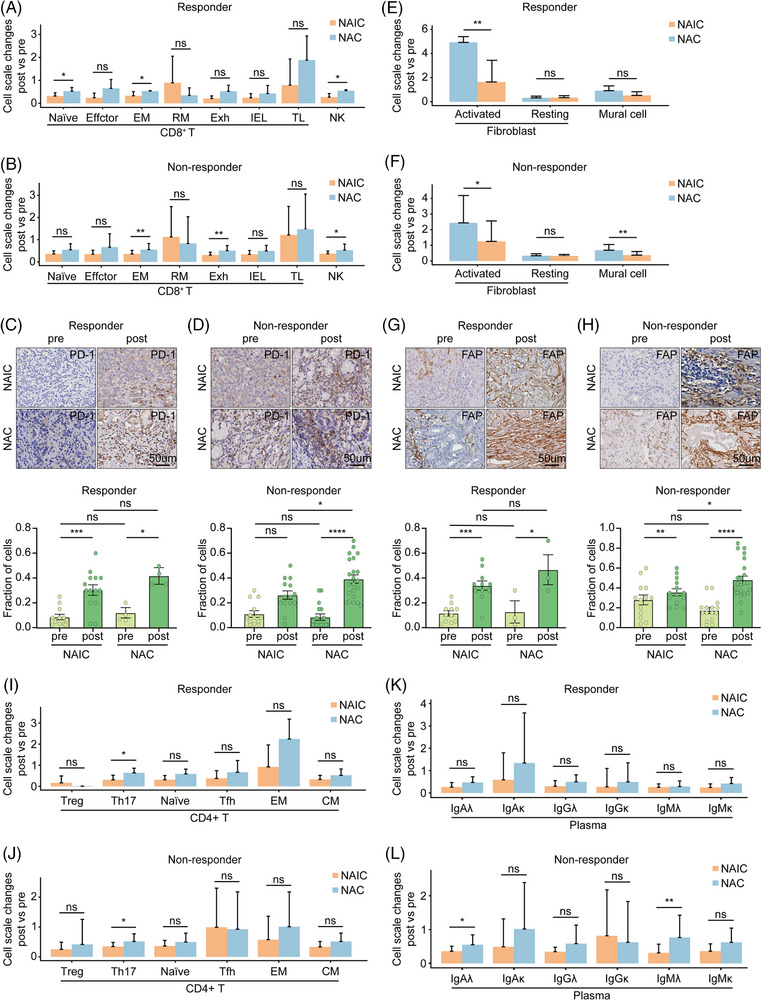
Dynamic changes of immune cell subsets during neoadjuvant immunochemotherapy and chemotherapy with different neoadjuvant efficacies. (A and B) Changes (post‐treatment proportions/pre‐treatment proportions) in different cell subtypes of CD8^+^ T cells under different neoadjuvant treatment regimens in the responder group (A) and the non‐responder group (B). (C and D) Representative images of PD‐1 staining of tumor specimens pre‐treatment (left) and post‐treatment (right) with different neoadjuvant treatment regimens (top). Quantification of exhausted T cells (PD‐1^+^ cells) by singlet immunohistochemistry and association with pre‐ and post‐treatment with different neoadjuvant regimens (bottom) at responder group (C) and non‐responder group (D). (E and F) Changes (post‐treatment proportions/pre‐treatment proportions) in different cell subtypes of fibroblasts under different neoadjuvant treatment regimens in the responder group (E) and the non‐responder group (F). (G and H) Representative images of FAP staining of tumor specimens pre‐treatment (left) and post‐treatment (right) with different neoadjuvant treatment regimens (top). Quantification of activated fibroblast cells (FAP^+^ cells) by singlet immunohistochemistry and association with pre‐ and post‐treatment with different neoadjuvant regimens (bottom) at responder group (G) and non‐responder group (H). (I and J) Changes (post‐treatment proportions/pre‐treatment proportions) in different cell subtypes of CD8^+^ T cells under different neoadjuvant treatment regimens in the responder group (I) and the non‐responder group (J). (K and L) Changes (post‐treatment proportions/pre‐treatment proportions) in different cell subtypes of plasma under different neoadjuvant treatment regimens in the responder group (K) and the non‐responder group (L).

We next investigated the dynamics of each cell type in relation to the efficacy of neoadjuvant therapies. Among the CD8^+^ T cell and NK cell types, we found that the increase in exhausted CD8^+^ T cells was significantly different only in the non‐responder group, suggesting that poor or no response to NAC may be associated with an increase in exhausted CD8^+^ T cells in the TIME (Figure 5A,B). These results were further validated by single‐cell immunohistochemistry of PD‐1 expression in exhausted CD8^+^ T cells (Figure 5C,D). Regardless of the degree of response to either neoadjuvant, both treatments induced fibroblast production, but the proportion of fibroblasts induced by NAIC treatment was significantly lower compared with NAC alone (Figure 5E,F). Immunohistochemistry analysis further elucidated that both neoadjuvant treatments resulted in an increase in fibroblasts in the TIME, but NAC had a higher proportion of fibroblasts than NAIC in the non‐responder group (Figure 5G,H).

In addition, we observed that Helper17 CD4^+^ T cells were significantly higher in the NAC group compared to in the NAIC group, regardless of response to neoadjuvant treatments (Figure 5I,J). On the other hand, significantly higher infiltration of IgAλ and IgMλ was observed in the NAC group compared to the NAIC only in the non‐response group. No significant difference was found between the two neoadjuvant treatments in the response group (Figure 5K,L). This result indicated that NAIC may be superior to NAC because it prevents the establishment of immunosuppressive cells such as exhausted CD8^+^ T and activated fibroblasts in the TIME.

### 2.6 Neoadjuvant immunochemotherapy promotes the formation and maturation of tertiary lymphoid structures

**FIGURE 6:**
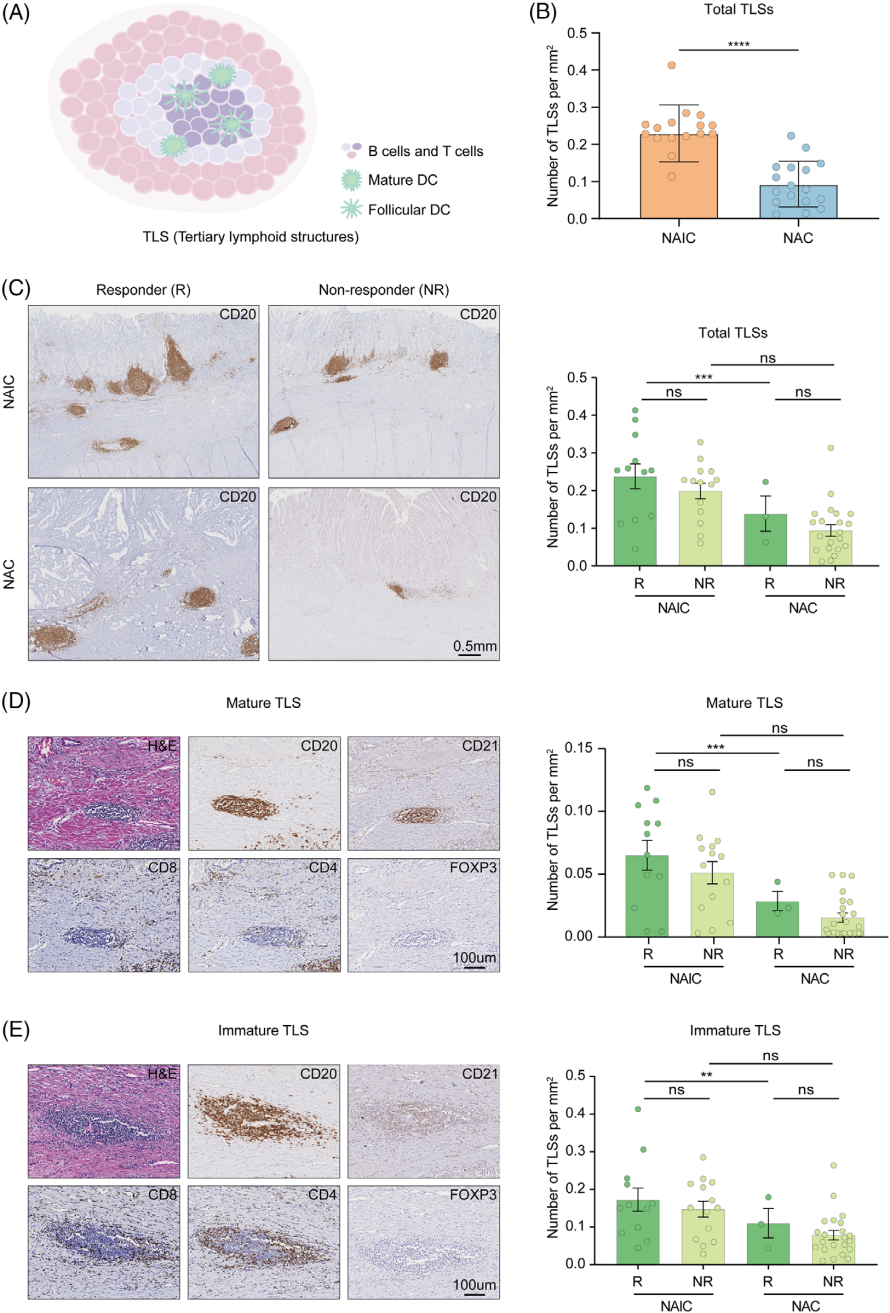
Maturation of tertiary lymphoid structures (TLSs) is induced by immunochemotherapy but not chemotherapy. (A) Putative model of TLSs. (B) Density of TLSs and correlation with different neoadjuvant regimens. (C) Representative image of CD20 staining of TLSs in responders (left) and non‐responders (right) of the neoadjuvant immunochemotherapy (NAIC) (top) or chemotherapy (bottom) group (left side). Density of TLSs and correlation by treatment response with different neoadjuvant regimens (right side). (D) Representative image of hematoxylin and eosin (H&E) staining and singlet immunostaining of CD20, CD8, CD4, FOXP3, and CD21 of mature TLSs (CD20^+^CD21^+^) (left side). Density of mature TLSs and correlation by treatment response with different neoadjuvant regimens (right side). (E) Representative image of H&E staining and singlet immunostaining of CD20, CD8, CD4, FOXP3, and CD21 of immature TLSs (CD20^+^CD21^−^) (left side). Density of mature TLSs and correlation by treatment response with different neoadjuvant regimens (right side).

Tertiary lymphoid structures (TLSs), which is formed in non‐lymphoid organs under chronic inflammatory stimulation including tumor, is mainly characterized by the aggregation of B cells and plays a key role in neoadjuvant cancer therapy (Figure 6A).^25^ Its presence is considered an effective indicator for predicting the response of patients receiving ICIs.^26^ We studied the tumor tissues of patients with locally advanced GAC who received NAIC or chemotherapy alone, using a singlet immunohistochemistry for CD20 to assess the presence of TLSs in gastric cancer tissues. Furthermore, we found that the abundance of TLSs in the TIME induced by NAIC was significantly higher compared with that of chemotherapy alone (Figure 6B).

We conducted a detailed analysis to examine the effects of immunochemotherapy and chemotherapy on the abundance and maturation of TLSs, as well as the association between TLSs abundance and maturation and pathological response. Among patients responding to neoadjuvant therapy, density of TLSs were significantly higher in patients receiving NAIC compared to chemotherapy alone. The analysis of TLSs showed that the number of these structures was higher in patients receiving NAIC compared to those receiving chemotherapy alone. This difference, although not significant, suggests that immunochemotherapy may promote the formation of TLSs in non‐responders (Figure 6C).

Mature TLSs were marked by CD21^+^ follicular dendritic cells, which show evidence of germinal center formation, suggesting the existence of an active B‐cell‐driven humoral antitumor response in the TLSs.^25^ Notably, analysis of TLSs revealed that not only CD20^+^ B cells were present in the TLSs of the tumor, but they also colocalized with CD4^+^, CD8^+^, and FOXP3^+^ T cells. According to the maturation of TLSs, we classified patients into the mature group (CD20^+^ CD21^+^, Figure 6D) and immature group (CD20^+^ CD21^−^, Figure 6E). Mature TLSs were also found to have similar results in our analysis, indicating that NAIC not only promotes the formation of TLSs, but also contributes to their maturation compared to chemotherapy alone. Importantly, it is significant to note that the number and maturity of TLSs are correlated with the efficacy of NAIC.

### 2.7 Activation of TNFα/NF‐κB signaling pathway serves as a unique molecular feature associated with treatment response in immunochemotherapy

**FIGURE 7:**
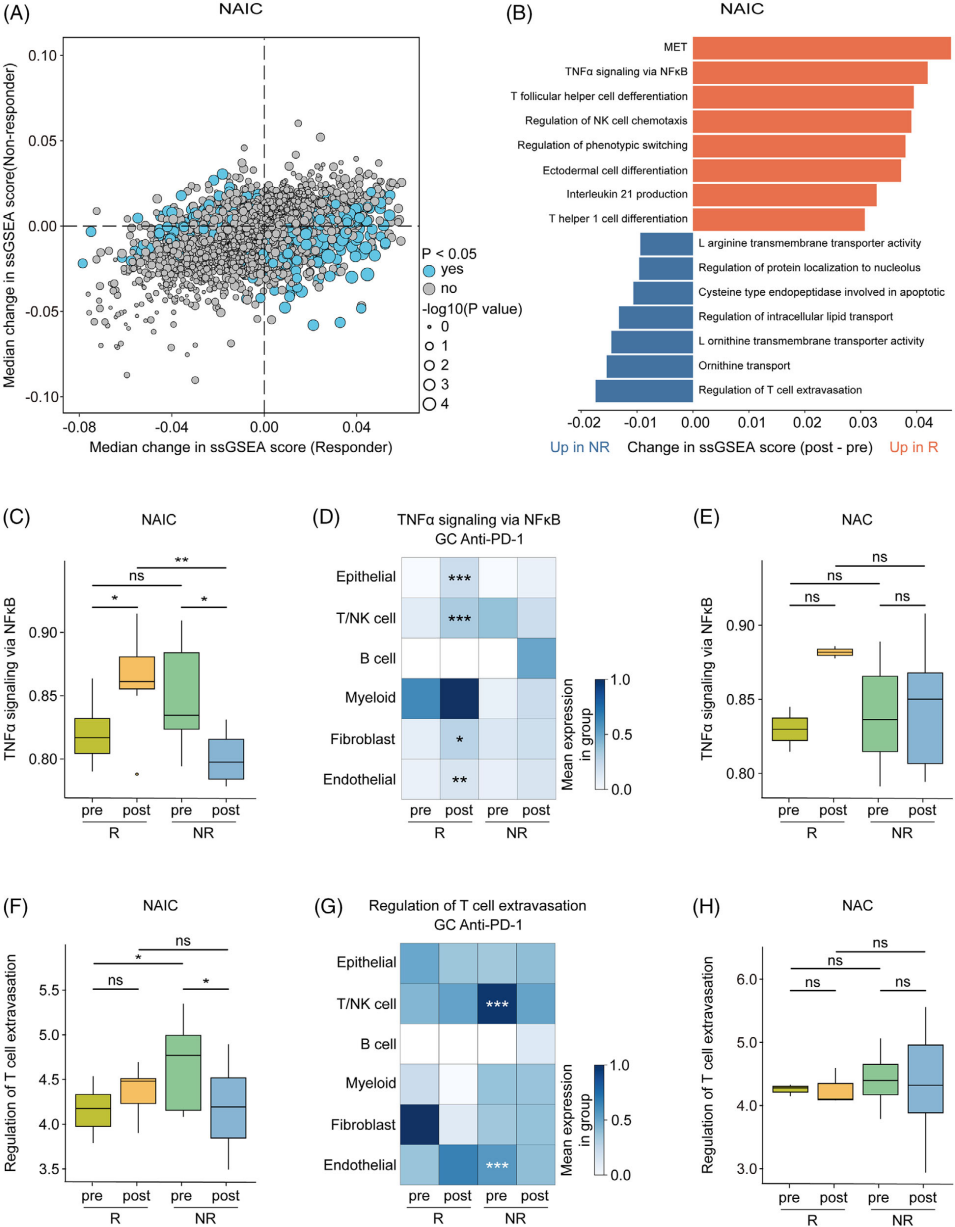
Transcriptomic analysis reveals therapy‐induced changes. (A) Hallmark and Gene Ontology resource (GO) gene sets median changes (post‐treatment vs. pre‐treatment) between responder group and non‐responder group during neoadjuvant immunochemotherapy (NAIC) treatment in paired samples. Significant pathways are highlighted in blue (*p* < 0.05). (B) Pathway enrichment results showing significantly enriched hallmark and GO gene sets in NAIC. Bar length represents median change in single‐sample gene set enrichment analysis (ssGSEA) score (post‐treatment vs. pre‐treatment); orange represents post‐treatment score increase in the NAIC responder group; blue represents the score decrease after treatment in the NAIC non‐responder group. (C) Changes in tumor necrosis factor alpha (TNFα) signaling via nuclear factor‐kappa B (NF‐κB) signatures before and after treatment in the NAIC group with different response groups. (D) Heatmap illustrating changes in TNFα/NF‐κB signaling in different cell types in gastric adenocarcinoma (GAC) treated with anti‐PD‐1 immunotherapy. Asterisks (*) represent significant changes after treatment in the anti‐PD‐1 responder group. (E) Changes in TNFα signaling via NF‐κB signatures before and after treatment in the neoadjuvant chemotherapy (NAC) group with different response groups. (F) Changes in regulation of T‐cell extravasation signatures before and after treatment in the NAIC group with different response groups. (G) Heatmap illustrating changes in regulation of T‐cell extravasation signatures in GAC treated with anti‐PD‐1 antibody. Asterisks (*) represent significant changes between the responders versus non‐responders in the baseline. (H) Changes in regulation of T‐cell extravasation signatures before and after treatment in the NAC group with different response groups.

To explore the molecular characteristics underlying the discrepancy in efficacy between two neoadjuvant treatment regimens. We initially assessed the differential transcriptomic effects between responders and non‐responders in NAIC by single‐sample gene set enrichment analysis (ssGSEA) of hallmark and Gene Ontology resource gene sets. The majority of pathways were significantly upregulated specifically in one group or the other (responder or non‐responder), and only a small number of pathways had significant changes that did not correlate with NAIC (Figure 7A). Changes in ssGSEA differed in responder compared with non‐responder. Responder upregulated, whereas non‐responder downregulated, pathways including mesenchymal‐to‐epithelial transformation, TNFα signaling via NF‐κB, and other immune‐related pathways. In addition, non‐responders increased expression of regulation of T‐cell extravasation, and some metabolism‐related pathways (Figure 7B). By contrast, the pathways related to the response to NAC include regulation of response to drug, meiotic cytokinesis, and morphogenesis of an epithelial fold. Pathways associated with non‐response to NAC are those related to immune processes mediated by MHC molecules and cell adhesion, among others (Figure S1A,B). Through ssGSEA analysis, we found that patients with locally advanced GAC have different response mechanisms when receiving NAIC or chemotherapy alone, and that NAIC enhances response more through the activation of immune and inflammation‐related signaling pathways.

Finally, we investigated whether NAIC‐related pathways are treatment‐unique. As expected, the TNFα/NF‐κB signaling pathway was significantly higher in responder patients compared to non‐responder patients at post‐treatment levels in the NAIC group (Figure 7C). To further explore the relationship between the TNFα/NF‐κB signaling pathway and response to immunotherapy at cellular levels, publicly available scRNA‐seq datasets of GAC samples following anti‐PD‐1 immunotherapy were also utilized, including nine samples from five GAC patients treated with immunotherapy, with a total of 39,818 cells were clustered into six cell types (Figure S1C,D). We found that in the responder group, the TNFα/NF‐κB signaling pathway was activated in epithelial cells, T cells, NK cells, fibroblasts, and endothelial cells (Figure 7D), suggesting that activation of TNFα/NF‐κB signaling in these cellular subsets may contribute to responsiveness to checkpoint inhibitors. In contrast, no significant changes were observed in the NAC group (Figure 7E), suggesting that the TNFα/NF‐κB signaling pathway could serve as a unique molecular maker for the response to NAIC. Furthermore, T‐cell extravasation signaling was elevated in T/NK cells and endothelial cells from pre‐treatment samples of the non‐responders to NAIC, as compared to the responders (Figure 7F,G). Whereas, no such changes were observed following NAC, suggesting that activation of the T‐cell extravasation regulatory pathway in T/NK cells and endothelial cells may predispose to non‐responsiveness to checkpoint blockade therapy (Figure 7H).

## 3 DISCUSSION

The present multi‐center cohort study demonstrates that NAIC achieves a higher pCR rate compared to chemotherapy. Together with observation from several recent reports^27^, ^28^, ^29^ and preliminary results from a prospective phase II trial (NEOSUMMIT‐01),^30^ these findings provide compelling evidence to support that NAIC may be an optimized approach to treat locally advanced GAC. Notably, patients with resectable gastric or gastroesophageal cancer were randomly assigned to receive either three cycles of SOX/XELOX or toripalimab plus SOX/XELOX, followed by five cycles of postoperative NAIC or NAC and tolipalizumab monotherapy for 6 months, NAIC significantly improved pathological regression compared with chemotherapy alone (MPR, 44.4% vs. 20.4%).^31^ Although NAIC achieved higher pathological regression compared with chemotherapy alone, a substantial portion of patients still do not experience benefits. Whether patients can achieve a survival benefit in the future still requires more clinical evidence from long‐term follow‐up. Similarly, there is still a need to further explore potential biomarkers, including combined positive score (CPS) score, microsatellite instability, and epstein‐barr virus (EBV) status, for predicting the efficacy of NAIC and to optimize personalized election.^32^

This study was the first to report that pretreatment intestinal subtype of Lauren's classification correlates with pathological regression only in the NAIC but not NAC, suggesting it could serve as a potential biomarker for patient stratification in the neoadjuvant treatment setting. Previous studies have demonstrated that phenotypic plasticity have been proposed for tumorigenesis, progression, and therapeutic resistance.^33^, ^34^, ^35^, ^36^ Here, we further show that NAIC elicits conversion of gastric malignant epithelial cells from intestinal to diffuse type, and this histological transition is associated with treatment resistance. We confirm that immunochemotherapy reduced intestinal‐to‐diffuse conversion, which is consistent with the clinical observation that NAIC led to significantly higher rates of pCR than chemotherapy alone.^31^, ^37^ Treatment‐induced phenotypic plasticity leading to treatment resistance has been reported in several cancer types. Detailed multi‐omics characterization of clinical samples with combined lung adenocarcinoma/small cell lung cancer histologic features and samples pre‐/post‐histologic conversion elucidates phenotypic plasticity as a mechanism of treatment resistance.^38^ Chemotherapy leads to semi‐squamatization in muscle‐invasive bladder cancer is associated with treatment resistance.^39^, ^40^ However, GAC is spatially highly heterogeneous. Accurate histological diagnosis relies on comprehensive review of all the tissue sections of the surgically resected tumor samples. The diagnosis of Lauren's classification of the pre‐treatment tumor samples obtained by endoscopic biopsy might lead to bias to some extent. With this notion in mind, our data suggested that reduced intestinal‐to‐diffuse conversion, a previously unrecognized type of phenotypic transition in GAC, is a unique feature of NAIC that is superior to chemotherapy alone.

The dynamic changes in epithelial and immunogenicity induced by neoadjuvant therapy were systematically explored by organically integrating the Lauren's classification with the TIME of gastric cancer. The correlation between TIME subtypes and immunotherapy response in gastric cancer was further validated in our study.^20^ We have found that the immune and stromal cells composition of the TIME is closely related to the epithelial cell morphology and degree of differentiation in GAC. More importantly, by comparing multi‐omics data pre‐ and post‐treatment, we revealed the dynamic changes of TIME subtypes and key cell subpopulations within them during the neoadjuvant treatment. Our study demonstrates that NAIC induces a shift in the TIME toward immunotherapy‐responsive subtypes, consistent with previous reports.^19^

Of note, gastric cancer of the intestinal type has tubular or glandular cell tissues with tight adherent connections and less stromal components, whereas diffuse‐type gastric cancer has scattered cell organization with poor adherence and poorly differentiated cells.^41^, ^42^ Meanwhile, diffuse gastric cancer is a pathologic type with poor prognosis and few treatment options compared to intestinal gastric cancer.^43^ We found that NF‐κB‐mediated TNF pathway is a unique molecular feature of NAIC response, which can promote cell terminal differentiation and improve chemotherapy resistance. During phenotypic conversion of lung adenocarcinoma, patients who transformed to small cell lung cancer exhibit significant downregulation of the TNF pathway compared to patients who never‐transformed non‐small cell lung cancer.^38^ Previous studies have also demonstrated that the TNF pathway can improve chemoresistance in bladder and breast cancers by inducing cell differentiation.^39^, ^44^ However, the underlying mechanisms for this phenotypic transition need further investigation.

Several limitations should be acknowledged in our study. First, we used three different treatment regimens in this study, which may have introduced bias that may have influenced the study results. Second, whether the reason for the histological conversion is treatment‐induced phenotypic plasticity or clonal selection of pre‐existing poorly differentiated tumor cells that lack cohesion and glandular structure remains to be further investigated. In conclusion, NAIC achieved higher pathological regression compared to NAC for locally advanced GAC. Intestinal subtype of Lauren's classification could be a potential biomarker for patient selection. Mechanistically, NAIC was effective in preventing intestinal‐to‐diffuse conversion and reprogramming of the TIME to be immunosuppressive, which may underlie superiority of NAIC over NAC. Further well‐controlled multi‐center clinical trials with long‐term follow‐up are warranted to determine whether enhanced pathological regression with NAIC is associated with reduced disease recurrence and longer survival for patients with locally advanced GAC.

## 4 MATERIALS AND METHODS

### 4.1 Data source of patients

Clinical data for both NAIC and NAC groups were collected from patients treated for locally advanced GAC at the Army Medical Center of PLA and the First Affiliated Hospital of China Medical University from January 2017 to July 2023. It was noteworthy that the clinical trial data on NAIC were obtained from patients enrolled for a prospective clinical trial of tislelizumab in combination with chemotherapy for neoadjuvant treatment of locally advanced GAC initiated on September 1, 2022 (Ratification No. 2022, No. 223; ClinicalTrial.gov identifier: NCT05515796). Although the prospective trial is still ongoing, here we retrospectively collected available data to compare clinical efficacy between NAIC and NAC.

The inclusion criteria for the retrospective analysis were patients with pathological diagnosis of G/GEJ adenocarcinoma, radiological diagnosis of cT1‐2N+M0/cT3‐4bNanyM0, patients who had received NAIC or chemotherapy, and had undergone surgical treatment. Exclusion criteria were patients with undetermined metastatic disease, receipt of other systemic therapy before neoadjuvant therapy, history of malignancy other than gastric cancer within the past 5 years, or failure to undergo surgical treatment.

A total of 295 patients met the inclusion criteria, with 67 patients in the NAIC group and 188 patients in the NAC group, respectively. The NAIC regimen was tislelizumab (tislelizumab 200 mg, day 1, every 3 weeks) plus XELOX (capecitabine and oxaliplatin; capecitabine 1000 mg/m^2^ twice a day for 2 weeks; oxaliplatin 130 mg/m^2^, day 1, every 3 weeks). For HER2‐positive GAC, trastuzumab was added to the regimen (trastuzumab 6 mg/kg following an initial loading dose of 8 mg/kg), day 1, every 3 weeks). The NAC regimen was XELOX or SOX (S‐1 and oxaliplatin; S‐1, 40 mg twice a day for 2 weeks; oxaliplatin 130 mg/m^2^, day 1, every 3 weeks), while trastuzumab was added for the HER2‐positive GAC.

### 4.2 Assessments

TRG criteria from the National Comprehensive Cancer Network were utilized to assess the pathological response of the primary lesion to perioperative treatment. This evaluation was conducted using the surgical tissue sample.^11^ TRG 0 indicates a complete pathological response (pCR), while TRG 0/1 indicates MPR. The patients with TRG 0 or 1 were classified as responders, while those with TRG 2 or 3 were classified as non‐responders.

### 4.3 Propensity score matching

To minimize potential confounding and selection bias, several factors including age, gender, Eastern Cooperative Oncology Group, tumor site, degree of differentiation, Lauren classification, clinical T stage, and clinical N stage were balanced. Binary logistic regression was used to generate propensity scores for each subject, ensuring a 1:1 ratio for PSM. PSM was performed using the following parameters: a caliper value of 0.1 and a random number of 123,456. The Matchlt, tableone, cobalt packages in R 4.2.0 was applied for PSM.

### 4.4 Sample collection and processing

All samples were obtained with written informed consent from the participants prior to enrollment. The pre‐treatment samples were taken by endoscopic biopsy, while post‐treatment samples were collected from the surgical resected primary tumors. All fresh samples were processed immediately to preserve in RNAlater (Invitrogen, AM7021) and stored in liquid nitrogen in two institutional biospecimen banks (the Army Medical Center of PLA and the First Affiliated Hospital of China Medical University). Alternatively, some paraffin embedded tumor samples were collected for RNA sequencing if frozen samples were not available. All the collected samples were utilized for RNA sequencing, as well as H&E staining for histological diagnosis, and immunohistochemistry staining for immunological analysis. The RNA sequencing data from fresh and paraffin embedded samples were analyzed using sva package to remove the batch effects.

### 4.5 H&E and immunohistochemical staining

Formalin fixation and paraffin embedding (FFPE) tumor tissue slides were used to conduct H&E and immunohistochemistry staining. First, the tissues were immersed in 10% formalin for fixation, and then 4‐µm serial sections were utilized for histopathological studies. The sections underwent sequential deparaffinization and hydration processes, and subsequently, the activity of endogenous peroxidase was neutralized using 3% H_2_O_2_. Afterwards, antigen was retrieved by utilizing citrate buffer with a pH of 6.0, and the sections were blocked with bovine serum albumin (BSA). Finally, the sections were stained using rabbit or mouse anti‐human monoclonal antibodies against CD4 (Zsbio, ZA‐0519, 1:100), CD8 (Zsbio, ZA‐0508, 1:100), CD20 (Zsbio, ZM‐0039, 1:100), CD21 (Zsbio, ZA‐0525, 1:100), CD56 (Zsbio, ZM‐0057, 1:100), FOXP3 (CST, 98377, 1:100), PD‐1 (Zsbio, ZM‐0381, 1:50), FAP (Abcam, ab227703, 1:200), and PD‐L1 (Dako, 22C3).

The sections were incubated with the primary antibody at 37°C for 2 h. After washed by phosphate‐buffered saline (PBS) for three times, the Horseradish peroxidase (HRP)‐conjugated secondary antibody (ZSGB‐BIO, Beijing) were added and incubated at 37°C for 30 min. Diaminobenzidine (DAB) was used to reveal the color and hematoxylin was used to counterstain. Finally, KFBIO's ScanScope system is employed for scanning and digitizing slides. The results were assessed by two independent pathologists. Immunohistochemical staining of PD‐L1 expression was performed using the PD‐L1 IHC 22C3 pharmDx kit (Dako) on the Dako ASL48 platform. CPS was used to evaluate the expression of PD‐L1. Mismatch repair (MMR) and HER2 status were evaluated separately through immunohistochemical staining of MMR proteins (MLH1, MSH2, MSH6, and PMS2) and HER2 antibody; HER2(2+) samples were further assessed through fluorescence in situ hybridization (FISH); EBV status was also evaluated by FISH.

### 4.6 Tumor purity and Lauren's classification assessments

Tumor purity was estimated histopathologically as the visually detected percent of tumor cells to all cells in the sample slide. According to the Lauren classification,^41^ GACs are divided into intestinal, diffuse, and mixed subtypes. The intestinal subtype shows tubular or glandular cellular organization with tight adhesive junctions and less stromal components, whereas the diffuse subtype shows scattered cellular organization, poor adhesion, and poor cellular differentiation. The mixed type was described as the combination of these two features. Two pathologists reviewed the original diagnostic slides to make a diagnosis of Lauren‘s classification.

### 4.7 Tertiary lymphoid structures quantification

H&E and CD20 immunohistochemistry staining were used to evaluate TLSs.^25^ Based on whether there is a germinal center, TLSs were divided into two stages of maturation: mature TLS (CD20^+^/CD21^+^) lymphoid follicles, which are clusters of lymphocytes with germinal centers; and immature TLS (CD20^+^/CD21^−^) lymphoid aggregates, which are clusters of lymphocytes and plasma cells without germinal centers. TLSs were assessed by two independent pathologists who were blinded to the clinical data.

### 4.8 Transcriptomic sequencing

The Vazyme VAHTS Total RNA‐seq (H/M/R) Library Prep Kit for RNA‐seq library construction of 64 cases of frozen samples with high quality, the TruSeq RNA Exome Prep Kit for RNA‐seq library construction of 32 cases of frozen samples with low quality and FFPE samples. Qubit 3.0 fluorometer (Thermo Fisher Scientific) was used to measure the concentration of RNA libraries, Agilent BioAnalyzer (Agilent) was used to analyze their size distribution. Sequencing was performed on Illumina NovaSeq6000 platform.

### 4.9 Gene expression analysis

The raw RNA‐seq reads were aligned to the human reference genome hg38 using STAR (v2.7.7a)^45^ and sorted using Samtools (v1.3.1).^46^ Read counts were calculated using GENCODE^47^ annotated gene models via featureCounts (v2.0.1)^48^ software. To adjust for batch effects due to differences in library construction, we used sva (v3.46.0) R package^49^ to create a linear regression model and adjust for these effects.

### 4.10 Single‐cell sequencing data processing

A scRNA‐seq dataset of 24 tumor samples from patients with GAC^50^ were processed using CellRanger toolkit (version 3.1.0) provided by 10× Genomics. Gene expression levels were quantified using human reference genome (GRCh38). The Python‐based toolkit Scanpy^51^ (version 1.9.8) was used for downstream analysis. For each cells identified by CellRanger, we calculated the total number of detected genes, total number of UMI counts, and proportion of mitochondrial reads. A set of quality thresholds was applied to filter out low‐quality cells, including detection of 200−7000 genes, and less than 15% mitochondrial reads, resulting in a total cell number of 39,818 post‐filter cells that were used for clustering analysis. Harmony was used to correct batch effects in different samples. The Leiden algorithm^52^ (resolution = 0.5) was used to cluster cells to iteratively group cells together, and the major six cell clusters were determined by well‐established cell markers (Figure S1C,D).

### 4.11 TIME subtype and deconvolution analysis

Four TIME subtypes were identified following the methods described in a previous study.^20^ The TCGA‐STAD samples were used as a training set to classify the TIME subtypes of the RNA‐seq data in our study using the Molecular Functional Portrait tool and the KNN model. BayesPrism methods^24^ were used to deconvolute the transcriptome data of our study using the gastric cancer single‐cell dataset^53^ as a reference. In the deconvolution analysis, genes related to mitochondrial and ribosomal proteins were excluded to minimize noise. To further mitigate the effects of technical batch variations, a differential expression analysis using the pair‐wise *t*‐test was conducted between cell states from different cell types. This analysis aided in the identification of genes, reducing the impact of noise caused by technical batch effects.

### 4.12 Gene set enrichment

The GSVA package was used to perform ssGSEA on the hallmark gene set and the C5 gene ontology bioprocess set of the raw RNA‐seq count matrix.

### 4.13 Statistical analyses

Statistical analysis details mentioned throughout the article are using R 4.2.0 software. The chi‐square test or Fisher's exact test is performed to analyze the association between categorical variables in more than two categories, while the Mann‒Whitney *U*‐test is applied for non‐categorical values. In addition, other statistical tests are mentioned in the figure legend. Significance values correspond to *p*‐values, *q*‐values, or false discovery rate (FDR) as follows: ns ≥ 0.05, ^*^<0.05, ^**^<0.01, ^***^<0.001, ^****^<0.0001. Standard deviation is provided for continuous variables with a normal distribution and interquartile range is provided for continuous variables with an abnormal distribution.

## AUTHOR CONTRIBUTIONS

Lei Wang wrote the manuscript. Lei Wang, Linghong Wan, and Peng Gao collected, organized, and analyzed clinical data. Lei Wang, Yongying Hou, Linghong Wan, Wenkang Liu, Shuoran Tian, Mengyi Han, Shiyin Peng, Yuting Tan, Yuwei Pan, Qin Liu, Jinyang Li, Haihui Wen, Mengsi Zhang, and Zhong‐Yi Qin performed experiments. Xu Chen, Yuanfeng Ren, Tao Wang, and Xianfeng Li performed all computational biology analysis. Shu‐Nan Wang, Chuan Chen, and Dongfeng Chen provided technical support. Junyu Xiang, Mengxia Li, Fan Li, Zhenning Wang, and Bin Wang designed the research and interpreted data. Bin Wang supervised the study, edited the manuscript, and approved the submission. All authors have read and approved the final manuscript.

## CONFLICT OF INTEREST STATEMENT

The authors declare they have no conflicts of interest.

## ETHICS STATEMENT

The prospective study of NAIC for locally advanced gastric cancer included in this study was approved by the Ethics Committee of Army Medical Center of PLA (ClinicalTrial.gov identifier: NCT05515796). This study also received approval from the Ethics Committees of Army Characteristic Medical Center and The First Affiliated Hospital of China Medical University (Ratification no. 2023(57)). Informed consent was obtained from the participants before enrollment in the study.

## Supporting information



Supporting Information

## Data Availability

The accession code for the raw FASTQ files from this paper are Genome Sequence Archive for Human: HRA006247. For other de‐identified participant data, study and statistical analysis protocols, they are available upon reasonable request from the corresponding author. A scRNA‐seq dataset of GAC treated by anti‐PD‐1 antibody was available from the European Nucleotide Archive (PRJEB40416).^50^ Treatment‐naive GAC scRNA‐seq dataset was downloaded from GEO repository (GSE206785).^53^
