# Supplementary material for: Reduced intestinal‐to‐diffuse conversion and immunosuppressive responses underlie superiority of neoadjuvant immunochemotherapy in gastric adenocarcinoma
Source: MedComm (2020). 2024 Oct 28;5(11):e762. doi: 10.1002/mco2.762 (PMC11518689; doi:10.1002/mco2.762)
Supplement: Supplementary file 1 — Supporting Information [file MCO2-5-e762-s001.docx]

**Supplementary Information for**

**Reduced intestinal-to-diffuse** **conversion and immunosuppressive responses underlie superiority of neoadjuvant immunochemotherapy in gastric adenocarcinoma**

Lei Wang, Linghong Wan, Xu Chen, Peng Gao, Yongying Hou, Linyu Wu, Wenkang Liu, Shuoran Tian, Mengyi Han, Shiyin Peng, Yuting Tan, Yuwei Pan, Yuanfeng Ren, Jinyang Li, Haihui Wen, Qin Liu, Mengsi Zhang, Tao Wang, Zhong-yi Qin, Junyu Xiang, Dongfeng Chen, Xianfeng Li, Shu-nan Wang, Chuan Chen, Mengxia Li, Fan Li, Zhenning Wang, Bin Wang.

**Supplementary Figure 1**

**Supplementary Table S1-3**

**
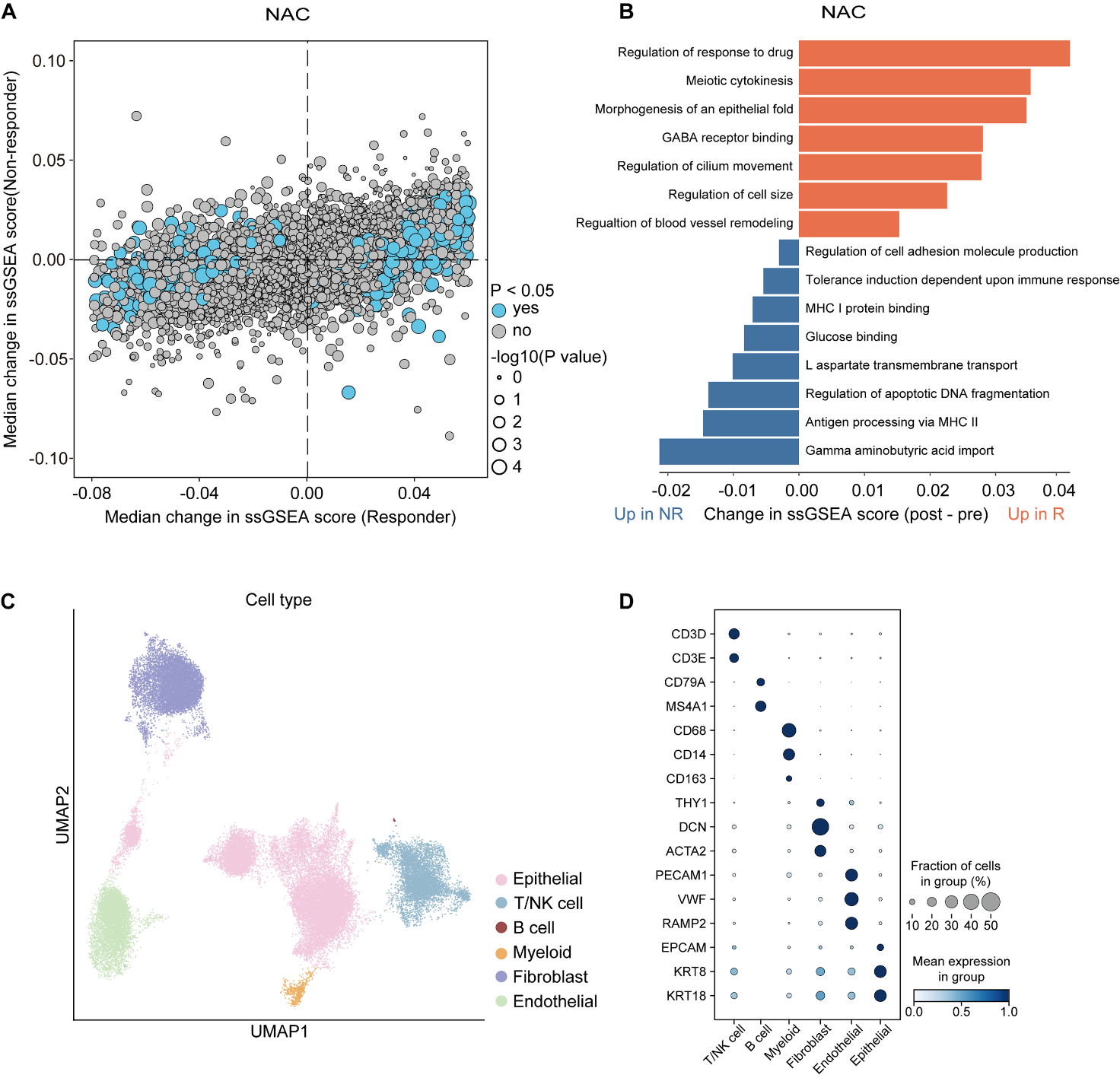
**

**Supplementary Figure 1. Transcriptomic analysis reveals therapy-induced changes in NAC and cellular landscape of GAC treated by anti-PD1 immunotherapy.**

A. Hallmark and GO gene sets median changes in the paired samples (post-treatment vs. pre-treatment) of the responders and non-responders following NAC treatment. Significant pathways are highlighted in blue (*P* < 0.05).

B. Pathway enrichment results showing significantly enriched hallmark and Gene Ontology resource (GO) gene sets in NAC. Bar length represents median change in ssGSEA score (post-treatment vs. pre-treatment); Orange represents post-treatment score increase in the NAIC responder group; Blue represents the score decrease after treatment in the NAIC non-responder group.

C. UMAP of 39,818 cells, color coded for six cell type.

D. Dotplot showing the scaled average expression together with the percentage of expression of marker genes.

Supplementary Table 1. Baseline characteristics

|  | **NAIC**, N = 63 | **NAC**, N = 63 | **p-value**^1^ |
| --- | --- | --- | --- |
| **Age, years, median (range)** | 62 (54, 68) | 61 (56, 67) | 0.8 |
| **Sex (n,%)** |  |  | 0.7 |
| FeMale | 22 (35%) | 20 (32%) |  |
| Male | 41 (65%) | 43 (68%) |  |
| **ECOG (n,%)** |  |  | 0.9 |
| 0 | 25 (40%) | 24 (38%) |  |
| 1 | 38 (60%) | 39 (62%) |  |
| **Tumor site (n,%)** |  |  | >0.9 |
| Antrum | 31 (49%) | 31 (49%) |  |
| Body | 15 (24%) | 14 (22%) |  |
| Cardia | 17 (27%) | 18 (29%) |  |
| **AJCC (n,%)** |  |  | 0.7 |
| I | 4 (6.4%) | 2 (3.2%) |  |
| II | 17 (27.0%) | 19 (30%) |  |
| III | 40 (63.4%) | 38 (60.4%) |  |
| IVA | 2 (3.2%) | 4 (6.4%) |  |
| **Histological Type (n,%)** |  |  | 0.4 |
| Adenocarcinoma | 57 (90.5%) | 54 (86%) |  |
| SRCC | 6 (9.5%) | 9 (14%) |  |
| **Differentiation (n,%)** |  |  | 0.8 |
| Well to Moderate | 3 (4.8%) | 4 (6.4%) |  |
| Moderate | 19 (30%) | 18 (28.6%) |  |
| Moderate to Poor | 14 (22.2%) | 18 (28.6%) |  |
| Poor | 27 (43%) | 23 (36.4%) |  |
| **Lauren (n,%)** |  |  | 0.8 |
| Intestinal | 22 (35%) | 21 (33%) |  |
| Mixed | 16 (25%) | 19 (30%) |  |
| Diffuse | 25 (40%) | 23 (37%) |  |
| **Clinical T category (n,%)** |  |  | 0.8 |
| 2 | 10 (16%) | 9 (14%) |  |
| 3 | 27 (43%) | 31 (49%) |  |
| 4 | 26 (41%) | 23 (37%) |  |
| **Clinical N category (n,%)** |  |  | 0.4 |
| 0 | 8 (13%) | 15 (24%) |  |
| 1 | 19 (30%) | 16 (25%) |  |
| 2 | 23 (37%) | 23 (37%) |  |
| 3 | 13 (20%) | 9 (14%) |  |

^1^Wilcoxon rank sum test; Pearson's Chi-squared test; Fisher's exact test

Supplementary Table 2. Surgical and pathology results of two treatment groups

|  | **NAIC**, N = 63 | **NAC**, N = 63 | **p-value^1^** |
| --- | --- | --- | --- |
| **TRG (n,%)** |  |  | 0.016 |
| 0 | 16 (25%) | 5 (8%) |  |
| 1 | 11 (17%) | 11 (17%) |  |
| 2 | 20 (33%) | 17 (27%) |  |
| 3 | 16 (25%) | 30 (48%) |  |
| **MPR (n,%)** | 27 (43%) | 16 (25%) | 0.039 |
| **R0 resection (n,%)** | 63 (100%) | 62 (98%) | >0.9 |
| **Gastrectomy (n,%)** |  |  | 0.2 |
| diatal | 32 (50%) | 23 (37%) |  |
| total | 26 (42%) | 35 (56%) |  |
| proximal | 5 (8%) | 5 (8%) |  |
| **Lauren (n,%)** |  |  | 0.024 |
| pCR | 16 (25%) | 5 (8%) |  |
| Intestinal | 10 (16%) | 9 (14%) |  |
| Mixed | 16 (25%) | 14 (22%) |  |
| Diffuse | 21 (34%) | 35 (56%) |  |
| **Vessel Invasion (n,%)** |  |  | 0.4 |
| Positive | 21 (33%) | 17 (27%) |  |
| Negative | 42 (67%) | 46 (73%) |  |
| **Nerve Invasion (n,%)** |  |  | 0.3 |
| Positive | 25 (40%) | 31 (49%) |  |
| Negative | 38 (60%) | 32 (51%) |  |
| **Pathological T stage post-surgery (n,%)** | |  | 0.038 |
| 0 | 17 (27%) | 5 (8%) |  |
| 1 | 4 (6%) | 5 (8%) |  |
| 2 | 9 (14%) | 11 (17%) |  |
| 3 | 15 (24%) | 26 (42%) |  |
| 4 | 18 (29%) | 16 (25%) |  |
| **Pathological N stage post-surgery (n,%)** | |  | 0.4 |
| 0 | 34 (55%) | 30 (48%) |  |
| 1 | 9 (14%) | 12 (19%) |  |
| 2 | 9 (14%) | 14 (22%) |  |
| 3 | 11 (17%) | 7 (11%) |  |
| **AJCC (n,%)** |  |  | 0.014 |
| pCR | 16 (24%) | 5 (8%) |  |
| I | 12 (17%) | 8 (13%) |  |
| II | 15 (22%) | 28 (44%) |  |
| III | 20 (29%) | 21 (33%) |  |
| IV | 0 (8%) | 1 (2%) |  |

pCR pathological complete responsem, MPR major pathological response.

^1^Pearson's Chi-squared test; Fisher's exact test

**Supplementary Table 3. Summary of adverse events**

|  | NAIC (N=63) | | NAC (N=63) | |
| --- | --- | --- | --- | --- |
|  | **Any grade** | **Grade3-4** | **Any grade** | **Grade3-4** |
| Treatment-related adverse events | 60(95.2%) | 17(27.0%) | 61(96.8%) | 19(30.2%) |
| Neutropenia | 30(47.6%) | 7(11.1%) | 29(46.0%) | 7(11.1%) |
| Thrombocytopenia | 15(23.8%) | 3(4.8%) | 14(22.2%) | 1(1.6%) |
| Leukopenia | 21(33.3%) | 4(6.4%) | 22(34.9%) | 4(6.4%) |
| Anemia | 12(19.0%) | 2(3.2%) | 13(20.6%) | 2(3.2%) |
| Nausea | 27(42.9%) | 1(1.6%) | 30 (47.6%) | 1(1.6%) |
| Vomiting | 7(11.1%) | 0 | 4(6.3%) | 0 |
| Diarrhea | 10(15.9%) | 0 | 10(15.9) | 0 |
| Alanine aminotransferase increase | 3(4.8%) | 0 | 8(12.7%) | 2(3.2%) |
| Aspartate aminotransferase increase | 3(4.8%) | 0 | 7(11.1%) | 2(3.2%) |
| Blood bilirubin increased | 2(3.2%) | 0 | 3(4.8%) | 1(1.6%) |
| Weight lost | 30(47.6%) | 3(4.8%) | 31(49.2%) | 4(6.4%) |
| fever | 1(1.6%) | 0 | 3(4.8%) | 2(3.2%) |
| Peripheral neuropathy | 8(12.7%) | 0 | 8(12.7%) | 0 |
| Hand foot syndrome | 7(11.1%) | 0 | 6(9.6%) | 2(3.2%) |
| Fatigue | 8(12.7%) | 0 | 11(17.5%) | 1(1.6%) |
| Immune-related TRAEs | 7(11.1%) | 0 | 0 | 0 |
| Hyperthyroidism | 1(1.6%) | 0 | 0 | 0 |
| Hypothyroidism | 2(3.2%) | 0 | 0 | 0 |
| Rash | 1(1.6%) | 0 | 0 | 0 |
| Hypopituitarism | 2(3.2%) | 0 | 0 | 0 |
| myocarditis | 1(1.6%) | 0 | 0 | 0 |
